# Validation and cultural adaptation of a German version of the Physicians' Reactions to Uncertainty scales

**DOI:** 10.1186/1472-6963-7-81

**Published:** 2007-06-11

**Authors:** Antonius Schneider, Joachim Szecsenyi, Stefan Barie, Katharina Joest, Thomas Rosemann

**Affiliations:** 1 Department of General Practice and Health Services Research, University Medical Hospital Heidelberg, Vossstrasse 2, 69115 Heidelberg, Germany; 2 Center of Psychosocial Medicine, Clinic of Psychiatry, University Medical Hospital Heidelberg, Vossstrasse 2, 69115 Heidelberg, Germany

## Abstract

**Background:**

The aim of the study was to examine the validity of a translated and culturally adapted version of the Physicians' Reaction to Uncertainty scales (PRU) in primary care physicians.

**Methods:**

In a structured process, the original questionnaire was translated, culturally adapted and assessed after administering it to 93 GPs. Test-retest reliability was tested by sending the questionnaire to the GPs again after two weeks.

**Results:**

The principal factor analysis confirmed the postulated four-factor structure underlying the 15 items. In contrast to the original version, item 5 achieved a higher loading on the 'concern about bad outcomes' scale. Consequently, we rearranged the scales. Good item-scale correlations were obtained, with Pearson's correlation coefficient ranging from 0.56–0.84. As regards the item-discriminant validity between the scales 'anxiety due to uncertainty' and 'concern about bad outcomes', partially high correlations (Pearson's correlation coefficient 0.02–0.69; p < 0.001) were found, indicating an overlap between both constructs. The assessment of internal consistency revealed satisfactory values; Cronbach's alpha of the rearranged version was 0.86 or higher for all scales. Test-retest-reliability, assessed by means of the intraclass-correlation-coefficient (ICC), exceeded 0.84, except for the 'reluctance to disclose mistakes to physicians' scale (ICC = 0.66). In this scale, some substantial floor effects occurred, with 29.3% of answers showing the lowest possible value.

**Conclusion:**

Dealing with uncertainty is an important issue in daily practice. The psychometric properties of the rearranged German version of the PRU are satisfying. The revealed floor effects do not limit the significance of the questionnaire. Thus, the German version of the PRU could contribute to the further evaluation of the impact of uncertainty in primary care physicians.

## Background

Dealing with uncertainty is a core competence for family physicians [1]. The impact of diagnostic uncertainty for primary care can be described using the Bayesian Theorem, which indicates that the positive predictive value of a diagnostic test for a disease is lower if the prevalence of this disease in the population is low [2]. Besides prevalence, another common cause of diagnostic uncertainty is that patients often see a doctor in the first stage of a disease where the symptoms are less distinct than in advanced stages [3]. Therefore, these unselected patient groups induce multiple decisional opportunities for diagnosing. The optimal treatment choice often appears to be uncertain in the light of increasing medical and technical progress which makes it nearly impossible for the individual doctor to keep an overview of newest developments in specific areas of diseases [4].

These problems are addressed by the implementation of guidelines [5] and the development of communication strategies to involve patients in treatment decisions [6]. However, individual attitudes and personal factors which influence a physician's ability to deal with uncertainty can hardly be targeted with these strategies. This is important because inadequate dealing with uncertainty may not only lead to higher costs but also harm patients [7]. The sources of uncertainty have been conceptualised in different ways [8], but its impact on clinical management remains widely elusive. Gerrity et al. developed a questionnaire for the conceptualisation and measurement of personal sources of uncertainty [9] which was revised in 1995 [10]. Allison et al. could demonstrate with this questionnaire that higher uncertainty is associated with higher resource use in a Medicare HMO [11]. Additionally, it could be shown that medical students with higher tolerance of uncertainty are more likely to choose careers such as primary care physicians whereas lower tolerance is more associated with urology or surgery [10,12]. Thus it seems worthwhile to explore the different influences of personal sources of uncertainty in medical care. Since there is no German questionnaire to measure physicians' reactions to uncertainty, the aim of this study was to evaluate the validity of a translated and culturally adapted version of the 'Physicians' Reaction to Uncertainty scales' (PRU).

## Methods

### Participants

The questionnaire was distributed to 93 GPs during a conference of the Department of General Practice and Health Services Research at the University Hospital in Heidelberg. GPs received the questionnaire together with short information about the aim of the study and were asked to personally complete the questionnaire. After two weeks, follow-up questionnaires were sent out to all GPs together with an explanatory note, saying that they should not try to remember their initial replies when answering the questionnaire for the second time. Sixty-four physicians (69%) returned their questionnaires.

### Translation and cultural adaptation

The German version of the PRU questionnaire was translated and back-translated according to guidelines for cultural adaptation in order to achieve the highest possible content validity [13]. The revised version of the Physicians' Reactions to Uncertainty scales comprised four scales, namely 'anxiety due to uncertainty' (items 1–5), 'concern about bad outcomes' (items 6–8), 'reluctance to disclose uncertainty to patients' (items 9–13) and 'reluctance to disclose mistakes to physicians' (items 14–15) [10]. The items are given in table 1. The items are rated on a 6-point Likert scale: 1 = strongly disagree, 2 = moderately disagree, 3 = slightly disagree, 4 = slightly agree, 5 = moderately agree, 6 = strongly agree. The scales are scored by summing physicians' response to each item in the scale (items 4, 9, 10 and 12 are reverse scored). The greater the score for a scale the greater the anxiety, concern about bad outcomes, reluctance to disclose uncertainty to patients, and reluctance to disclose mistakes to physicians, respectively. Slight adaptations were necessary for items one and twelve. This was done to obtain a more understandable translation which captured the original idea of the item rather than for the more direct translation. For example 'anxious' is better captured with the German translation of 'worried' (item one). 'Sharing my uncertainty' could only be reasonably translated with the German translation of 'inform about my uncertainty' (item twelve). The draft translation was piloted with 15 GPs.

**Table 1:** Principal component factor analysis with varimax rotation (eigenvalue > 1)

	**Anxiety due to uncertainty**	**Concern about bad outcomes**	**Disclosing uncertainty to patients**	**Disclosing mistakes to physicians**
**Explaining % of variance**	19.88	20.31	18.57	12.97

1. I usually feel anxious when I am not sure of a diagnosis.	**0.757**	0.198	0.147	-0.018
2. I find the uncertainty involved in patient care disconcerting.	**0.858**	0.259	0.048	0.118
3. Uncertainty in patient care makes me uneasy.	**0.844**	0.301	0.084	0.032
4. I am quite comfortable with the uncertainty in patient care.	**0.727**	0.167	0.093	0.165
5. The uncertainty of patient care often troubles me.	0.442	**0.690**	-0.025	-0.007
6. When I am uncertain of a diagnosis, I imagine all sorts of bad scenarios – patient dies, patient sues, etc...	0.186	**0.824**	-0.001	-0.015
7. I fear being held accountable for the limits of my knowledge.	0.211	**0.853**	-0.102	0.070
8. I worry about malpractice when I do not know a patient's diagnosis.	0.279	**0.851**	0.026	0.112
9. When physicians are uncertain of a diagnosis, they should share this information with their patients.	-0.042	0.058	**0.709**	0.299
10. I always share my uncertainty with my patients.	0.165	-0.235	**0.774**	-0.022
11. If I shared all of my uncertainties with my patients, they would lose confidence in me.	0.118	0.371	**0.618**	-0.077
12. Sharing my uncertainty improves my relationship with my patients.	0.059	-0.114	**0.829**	0.196
13. I prefer patients not know when I am uncertain of what treatments to use.	0.088	-0.008	**0.728**	-0.002
14. I almost never tell other physicians about diagnoses I have missed.	0.098	0.052	0.145	**0.935**
15. I never tell other physicians about patient care mistakes I have made.	0.124	0.046	0.102	**0.936**

### Statistical analysis

Data were entered using Microsoft Excel spreadsheets and analysed with the SPSS statistical package (version 12.0). When necessary, items were recoded and transformed from graded 6 point scales (according to the recommendations of Gerrity et al. [10]). Descriptive analysis included mean, standard deviation and, in order to assess floor and ceiling effects, the percentage of participants achieving the lowest and highest possible score.

#### Construct validity

To explore the construct validity of the four constructs underlying the 15 items, we conducted a principal component factor analysis with varimax rotation. The criterion for factor extraction was an eigenvalue >1.0.

#### Internal consistency

To assess internal consistency we calculated Cronbach's alpha to estimate the correlation of each item with the underlying concept of its scale [14,15]. Achievable values for Cronbach's alpha range from 0 (signifying no correlation) to 1 (indicating identical results). We adopted the position that an acceptable reliability constitutes Cronbach's alpha ≥ 0.70 [15].

#### Scale internal validity

Scale internal consistency was assessed by calculating the correlation of the items with the respective scale corrected for overlap to avoid the bias of self-correlation (Pearson's correlation coefficient). A correlation coefficient of ≥ 0.4 was used as a standard for assuming good scale-internal consistency.

Item-discriminant validity shows the extent to which an item measures what it is not supposed to measure. Therefore, the item-discriminant validity indicates the degree of discriminatory power. It is assessed by calculating the correlation of the items with the scales which they are not grouped into (Pearson's correlation coefficient). Cut-off values have not been defined, but in order to support high discriminatory power of scales, the correlation of an item with the other non-item scales should be low to achieve satisfying discriminance.

#### Test-retest-reliability

We used the intraclass correlation coefficient (ICC) as an estimate of test-retest-realibility of the individual scales. We calculated the ICC based on the four components model of Gerrity et al. [10]. The calculation was performed with the sixty-four questionnaires (69% of 93) which were returned after two weeks.

## Results

### Descriptive analysis

All of the initially distributed questionnaires and 64 (69%) of the redistributed questionnaires to assess test-retest-reliability were returned. As displayed in table 2, there were more male participants; and they were older than the female participants (p < 0.001). Overall, professional experience was high with a mean of 21.0 years working as a physician and 14.4 years working as a GP in practice. There were no significant differences related to sex or age between responders and non-responders of the retest evaluation (not in table).

**Table 2:** Baseline characteristics

**Sex**	**n**	**Years in private practice**	**Years of clinical experience**	**Age**
		**Mean (SD)**	**Mean (SD)**	**Mean (SD)**
Female	25	9.1(6.7)	15.3 (5.5)	44.2 (6.1)
Male	68	15.8 (8.3)	23.0 (8.2)	50.8 (7.7)
Total	93	14.4 (8.4)	21.0 (8.3)	49.2 (7.9)

Table 3 shows the descriptive statistics for the individual scales. The answers covered the full range indicating the usefulness of the scaling. A substantial floor effect occurred in the scale 'reluctance to disclose mistakes to physicians'. Notable ceiling effects did not occur.

**Table 3:** Descriptive values of the diagnostic uncertainty questionnaire

**Scale**	**Range**	**Min**	**Max**	**Mean**	**Median**	**SD**	**Floor**	**Ceiling**
Anxiety	25.0	5.0	30.0	17.6	18.0	6.2	2.2%	2.2%
Bad outcomes	15.0	3.0	18.0	8.2	7.0	3.9	7.5%	2.2%
Disclose to patients	25.0	5.0	30.0	14.9	15.0	5.2	5.6%	1.1%
Disclose to physicians	10.0	2.0	12.0	4.1	4.0	2.2	29.0%	1.1%

Table 1 shows the results of the factor analysis after varimax rotation with the four extracted factors. The factor 'anxiety due to uncertainty' explains 19.88% of the cumulated variation, the factor 'concern about bad outcomes' 20.31%, 'reluctance to disclose uncertainty to patients' 18.57% and the factor 'reluctance to disclose mistakes to physician' 12.97%. The high loading of the respective items related to the four factors confirms that the scales are clearly distinguished. The principal factor analysis revealed an interesting result: the factor loading for item 5, which was grouped in the scale 'anxiety due to uncertainty' in the original version was much higher for the scale 'concern about bad outcomes'. Consequently, we rearranged the scales according to the factor loading.

### Assessing scale internal validity and reliability

Since rearranging of scales can have substantial influence on reliability, we compared Cronbach's alpha of the original version and of the German version with and without rearrangement. Table 4 shows the scale internal validity and reliability of the questionnaire. As regards the scale internal consistency, the correlations of single items and the referring scale ranged from 0.56 to 0.84, indicating good item-scale correlations. The item-discriminant validity assessed by calculating correlations of the items with scales, they are not grouped into, revealed a comparatively high overlap between the items of the scales 'anxiety due to uncertainty' and 'concern about bad outcomes'. Pearson's correlation coefficient between item 3 (belongs to scale one) and scale two was 0.49. Pearson's correlation coefficient between item 8 (belongs to scale two) and scale one was 0.57, and it was 0.69 between item five (belongs to scale two in the German version) and scale one (p < 0.001). The comparatively high correlation between 'anxiety due to uncertainty' and 'concern about bad outcomes' also indicates a strong relation between these scales (Pearsons' correlation coefficient 0.59, p < 0.001; Table 5).

**Table 4:** Parameter of scale internal validity and reliability

	**Rearranged German version**				**German original**	**Gerrity**
**Scales**	**Item-scale-correlation**	**Item-discriminant validity**	**Test-retest correlation (ICC)**	**Reliability (Cronbach's alpha)**	**Reliability (Cronbach's alpha)**	**Reliability (Cronbach's alpha)**

Anxiety	0.58–0.83	0.15–0.49	0.85	0.866	0.86	0.86
Bad outcomes	0.67–0.81	0.02–0.69	0.90	0.874	0.86	0.73
Disclose to patients	0.56–0.71	0.03–0.32	0.84	0.86 (not rearranged)	0.86	0.79
Disclose to physicians	0.84	0.15–0.22	0.66	0.91 (not rearranged)	0.91	0.72

**Table 5:** Correlation matrix of PRU scales

**Scales**	**Bad outcomes**		**Disclose to patients**		**Disclose to physicians**	
	**corr**	**p**	**corr**	**p**	**corr**	**p**
Anxiety	0.59	<0.001	0.21	0.05	0.23	0.03
Bad outcomes	-		0.02	0.89	0.15	0.14
Disclose to patients	-		-		0.21	0.05

corr = Pearsons' correlation coeffecient

As indicated in table 4, the rearranged German version achieved higher values for Cronbachs's alpha not only compared to the original German version, but also when compared to the original version by Gerrity et al. [10]. In particular the Cronbach's alphas of the scales 'reluctance to disclose uncertainty to patients' and 'reluctance to disclose mistakes to physicians' were high when compared to Gerrity et al. [10]. The values for the test-retest-reliabilty, displayed as ICC are quite good, except for that of the scale 'reluctance to disclose mistakes to physicians'.

## Discussion

The translated version of the PRU questionnaire was evaluated using a sample of 93 primary care physicians. We received very satisfactory internal validity and reliability which partly surpassed the validation results of the original questionnaire [10]. The scales 'anxiety due to uncertainty' and 'concern about bad outcomes' improved by shifting the fifth item ('The uncertainty of patient care often troubles me') from the first to the second scale.

In the original questionnaire the scales 'concern about bad outcomes' and 'reluctance to disclose mistakes to physicians' had Cronbach's alpha values of 0.73 and 0.72 respectively [10]. These internal consistencies remarkably increased in the German version (0.87 and 0.91). This difference can possibly be explained by the different validation groups which comprised various specialities in the original version whereas our sample was a homogenous group of primary care physicians. Another reason might be a changed attitude due to a more open culture of discussion about medical errors and uncertainty in recent years. The original questionnaire was validated in 1992 [9] and revised in 1995 [10]. However, awareness of medical errors has increased internationally since the Institute of Medicine issued *To err is human *[16] in 1999. The reports which primarily focussed on adverse events in hospitals [17] expanded also into the field of family practice with time [18]. There is also an initiative for a web-based voluntary error reporting system established in Germany in 2004 which received a great deal of attention among German physicians [19]. Therefore it could be speculated that German physicians (and perhaps also physicians from other nations) nowadays have a greater directness in dealing with errors and disclosing mistakes to other physicians. The small but lower mean of that scale (mean 4.1) compared to the original questionnaire (mean 4.4 [10]) points in that direction. The lower mean in 'concern about bad outcomes' (8.2 compared to 9.5 [10]) might be due to the lower risk of being sued in Germany, which leads to more directness in answering the questions of this scale.

Interestingly, the reluctance to disclose uncertainty seems to be higher in our German study than in the study by Gerrity et al. (14.9 vs 13.6). This may be due to the communication style being more participatory in the US than in Germany. This could be hypothesized as the movement of shared decision making started in Anglophone countries [20] and is only slowly being adopted in Germany [21]. Further research is needed to compare different styles of dealing with uncertainty across different countries.

Another important aspect is the partly low item-discriminant validity between 'anxiety due to uncertainty' and 'concern about bad outcomes'. This point to some overlap of both psychological constructs. This is also indicated by the fact that the item 'The uncertainty of patient care often troubles me' loaded higher on the scale 'Concern about bad outcomes'. Obviously, German physicians seem to associate this statement more with serious consequences than with anxiety. Due to the stronger association, this item should be grouped into the second scale in the German version to improve the internal consistency. It would be worthwhile to perform factor analyses in further evaluations, possibly across different cultures, to evaluate the generalisability of this finding.

Some limitations of the study have to be noted. The high internal consistency might be due to the homogenous group of physicians we used to evaluate the questionnaire. Therefore, the German version of the PRU should be re-validated with specialized medical doctors to evaluate its transferability. Another limitation is that we could not evaluate the external validity as there is no similar questionnaire available in Germany. However, as we had similar results to those of Gerrity et al. [10] it seems reasonable to accept this cultural adaptation as a reliable instrument for measuring German physicians' reaction to uncertainty.

In general, this questionnaire could be used in several ways. As an example, personal sources of uncertainty could be identified and included in quality improvement projects. It has been shown that feedback of testing strategies improves test ordering in primary care [22-24]. However, it is widely accepted that not only doctors'[10,11,25] but also patients' characteristics[26] and the interaction [27] between them are important determinants of the management of medical uncertainty. The resulting uncertainties in medical management are supposed to be solved with tacit knowledge[28]. It remains unclear indeed, if test ordering behaviour could be changed if the ability to deal with uncertainty is analyzed and included in feedback or discussion within a physician group.

With increasing medical and technical progress, the demand for certainty increases, leading to a paradoxical intolerance of uncertainty in patients and doctors [8,29]. As uncertainty is particularly inherent in primary care[1,2] it might be one important reason for the lack of GPs, not only in Germany but also in other developed health care systems. It needs to be evaluated whether basic lessons in Bayesian reasoning, possibly combined with reflection on personal attitude, are able to strengthen tolerance towards uncertainty in medical care. As a consequence, motivation for being a GP could be enhanced. Therefore, the PRU could not only serve as an indirect measure of changing attitude but also provide a better understanding of the physicians' ability to deal with uncertainty.

## Conclusion

Diagnostic uncertainty is an important issue in daily practice. However, its impact and the way of dealing with uncertainty remain widely elusive. The PRU questionnaire could contribute to further evaluation. The psychometric properties of the rearranged German version of the PRU are satisfying. The revealed floor effects do not limit the significance of the questionnaire.

## Competing interests

The author(s) declare that they have no competing interests.

## Authors' contributions

AS designed the trial, performed the statistical analysis and wrote the manuscript. JS helped to write the manuscript. SB helped with data management and analysis. KJ helped to translate the questionnaire and with writing. TR helped to translate the questionnaire and with statistical analysis.

## Pre-publication history

The pre-publication history for this paper can be accessed here:


